# Use of medicinal plants by veterinary practitioners in Spain: A cross-sectional survey

**DOI:** 10.3389/fvets.2022.1060738

**Published:** 2022-12-15

**Authors:** Beatriz Romero, Julen Susperregui, Ana M. Sahagún, M. José Diez, Nélida Fernández, Juan J. García, Cristina López, Matilde Sierra, Raquel Díez

**Affiliations:** ^1^Department of Biomedical Sciences, Veterinary Faculty, Institute of Biomedicine (IBIOMED), University of León, León, Spain; ^2^Applied Mathematics, Department of Mathematics, University of León, León, Spain

**Keywords:** Spain, veterinarian, medicinal plants (herbal drugs), pets, use pattern

## Abstract

Medicinal plants have been used in veterinary medicine since ancient times, and they are gaining importance in Eastern Europe. The aim of this study was to conduct a survey on the use of medicinal plants in Spain. A cross-sectional study with an online questionnaire was carried out among Spanish small animal veterinarians, to evaluate the use patterns of medicinal plants and attitudes of professionals toward it. 313 veterinarians took part in the study. Most of them were female (80.2%) and age ranged 35–49 (49.5%). 80.3% of respondents use phytotherapy. Musculoskeletal and gastrointestinal disorders were those most frequently treated, with cannabis, aloe and thyme the most often medicinal plants used. The most common pattern of user was women working in clinics.

## Introduction

Traditional folk veterinary medicine is defined as a mode of identifying, using and integrating local knowledges, related skills and custom procedures created by people with the purpose of preserving animals' health and welfare (1). Those traditional drugs practices are scarce in Europe as medicinal plants have been replaced by modern medicine. However, in the last decades, the study and use of medicinal plants in veterinary medicine has gaining importance, especially in Italy, Germany or Eastern Europe (2–7), as some veterinarian practices, related to the use of medicinal plants, are still alive in these countries. Regarding Spain, medicinal plants is an underexplored field although it should be considered an alternative to improve animal health. Few studies (8–10) on plant-based ethnoveterinary knowledge have been carried out, mainly as catalogs and descriptions of those medicinal plants commonly used. Of note is the Spanish Inventory of Traditional Knowledge, which describes the use of 711 species of vascular plants as veterinary phytotherapy (11). This large number of plant species makes Spain a country with great wealth and potential for use in veterinary medicine. Carrió et al. (10) have already explored this possibility by comparing the potential uses of medicinal plants from Catalonia and the Balearic Islands in human and veterinary medicine.

There are several reasons to explain this increased attention to herbal medications in veterinary medicine. Among them, there is a widespread belief among population that medicinal plants are effective and, at the same time, safer than synthetic compounds. Another main reason is also economic, as they tend to be cheaper than conventional treatments. Moreover, they are considered a more sustainable approach (12). In this sense, phytotherapeutic remedies are also a viable option for organic livestock treatments, avoiding the use of synthetic drugs. In some cases, people seek outside the allopathic approach to meet healthcare needs, for chronic or untreatable diseases and, in others, some of these medicinal plants are used to strengthen the immune system (13). On the other hand, they may help to reduce the abuse of antibiotics and antiparasitics, which would minimize the transmission of resistant pathogenic bacteria through food consumption or due to direct animal contact (14). In addition, in spite of establishing maximum residue limits (MRL) and withdrawal periods, chemical compounds are not free of risk, and sometimes may persist in livestock meat, increasing the potential human exposure through food consumption, especially for those substances that can be accumulated in the human body (15).

Ethnoveterinary approach may also be framed within the One *Health* concept, which considers human and animal health and the environment as interconnected (14, 16, 17). Although the term One *Health* is relatively recent (18, 19), it has its origin in the so called One *Medicine* approach (18), which recognized as early as 1976 the close interaction between animals and human beings in health.

There are many animal species for which medicinal plant-based treatments are recommended. Most of the studies focused mainly on treatment of productive large animals by herbal medicines (6, 7, 12, 20, 21). In contrast, information on the use of medicinal plants in small animals is scarce. According to Viegi et al. (2), large animals such cattle, horses, sheep, goats and pigs represented 70.5% of domestic animals treated with herbal medicines in Italy, whereas dogs were only 5.3%. Regarding pets, the use of plant-based medicine is increasing in these animals due to its effectiveness and adequate benefit-risk balance, but also because it may be useful for treating subclinical diseases or those chronic ones without conventional treatment, as well as disorders for which there is no need of professional diagnosis (22), although it is not easy to establish the extent to which traditional treatments are used in veterinary clinics. In Western countries, veterinarians are initially trained in modern therapy, but other researchers have shown that the use of phytotherapy is not uncommon. In a survey carried out on the use of herbal medicines in small animals in German-speaking countries, approximately three quarters of veterinarians employed herbal medicines, especially for chronic diseases and as adjunct therapy (3), and in an academic teaching hospital a similar percentage (76.4%) of small animals was treated with herbal supplements (23).

Although the use of medicinal plants is increasing in pets, there are very few studies on its use, and especially in Spain, where no data have been found for companion animals. In this sense, we have detected a gap in the scientific literature regarding it in Veterinary Medicine. Thus, this study aims to determine the current status of this field in Spain, in an attempt of recording the use of medicinal plants as well as the opinion, attitudes, and degree of acceptance among the small animal practitioners. For this purpose, several predictor variables were evaluated to establish if they were associated with the use of phytotherapy in companion animals, to provide a comprehensive picture of the determinants or reasons governing the choice of this kind of treatment by practitioners.

## Materials and methods

### Research and questionnaire design

An observational and descriptive cross-sectional study was carried out among Spanish veterinarians. An online survey was carried out by using a cloud-based survey tool (Google Forms) (Supplementary Figure S1). The research team designed the questionnaire through a comprehensive literature review and the findings of previous studies (3, 24). The questions were selected in collaboration with veterinary researchers and practitioners. The questionnaire was written in Spanish and consisted of 15 questions, grouped into two sections. In the first section (5 questions), veterinarians were requested to provide sociodemographic information, such as gender, group of age, place of residence, workplace, and type of small animals treated (for the purpose of the study, small animals were grouped as dogs, cats and other animals including rabbits, rodents, birds and reptiles). In the second section (10 questions), they were requested to answer on the use of medicinal plants in their patient care activity. Multiple-choice questions with a single (8 questions) or multiple answer (5 questions), as well as 2 open-ended questions were included. All participants received the same questions in a fixed order. The questionnaire was previously provided to 5 practitioners to verify that it was well understood, and to check the time needed to answer it (~8 min). Practitioners were asked whether they had ever heard about phytotherapy, if they were for or against this type of therapy, if they have ever used medicinal plants and which ones, the diseases treated, and those dosage forms commonly employed. Moreover, the common and scientific names of these plants were included in the questionnaire, as they were easily recognizable by the clinicians.

Sample size was determined on the number of members (5,600) in the Spanish Small Animal Veterinary Association (AVEPA). Assuming a precision of 0.06, an estimated probability of 0.5 and a significance level of 0.05 (25), the necessary minimum sample size was estimated in 255 veterinarians.

The Strengthening the Reporting of Observational Studies in Epidemiology—Veterinary extension (STROBE-Vet) Statement was used to report data (26).

### Data collection

As explained, the questionnaire targeted companion animal practitioners throughout Spain. The survey was completed between March and September 2021. The questionnaire was available online to reach as many veterinarians as possible. Facebook social media was used to promote the survey among networking groups of practitioners and recruit participants. The status was checked on a 2-week basis, and four reminders were sent on social media. All veterinarians were required to have at least 1 year of clinical experience to take part, participated on a voluntary basis and gave their informed consent before starting the survey. The questionnaire was anonymous, answers were coded to maintain confidentiality, and participants were free to end the survey at any time they wished. Data collection, processing and storage was carried out in accordance with Spanish regulations (27). The study was approved in advance by the Ethics Committee of the University of Leon (ULE0382018), and carried out in accordance with the Declaration of Helsinki.

### Data analysis

Once data were obtained, they were processed and analyzed using Microsoft Office Excel (2016) and SPSS Statistics 26 (IBM Corporation, Armonk, NY, USA). Internal consistency and reliability were assessed using McDonald's omega (28). Descriptive statistics (frequencies, median, standard deviations, ranges, and percentages with 95% confidence intervals) were used to characterize the study population. Chi-square (χ^2^) and the Fisher's exact tests were employed to compare qualitative variables (gender, group of age, workplace). The first one was used to test proportions and, if samples were small, the Fisher's exact test. For quantitative variables (number of health problems, number of natural products and number of pharmaceutical forms), comparisons were made with Kruskal-Wallis (more than two groups) or Mann-Whitney *U* tests (two groups). Logistic regression was also performed to identify those demographic variables (gender, group of age, workplace and type of small animals treated) potentially associated with the use of medicinal plants. The odds ratio (OR) was calculated with their respective 95% confidence intervals (95% CI). Differences were always considered significant with *p* ≤ 0.05.

## Results and discussion

A total of 313 valid responses were received, which exceeds the minimum sample size required. A value of 0.86 was calculated for McDonald's omega, showing a good reliability and internal consistency.

Most of the respondents were female practitioners (80.2%). 49.5% aged 35–49, one third (32.6%) were aged 24–34 and the rest were in the 50–64 age group (17.9%). In 2020, a total of 34,443 veterinarians were registered in Spain, and 50.9% were female (29). Regarding gender and age, female frequency was similar in the age groups 24–34 years (85.3%) and 35–49 (83.2%), with no significant differences, whereas female proportion was significantly lower in group ≥50 years (62.5%) (χ^2^ = 13.604; *p* = 0.001).

As for the place of residence, most participants lived in Madrid, Leon and Barcelona, which are also the geographical regions with the highest rate of veterinarians registered in this country. According to the Spanish Institute of Statistics (INE), in 2020 11.7% of the Spanish veterinarians were registered in Madrid, 8.0% in Barcelona and 2.9% in Leon (30). Most of them worked in a veterinary clinic (88.5%), which is also in accordance with *Veterinary Management Studies* (31) in 2019, as 71% of the 6,228 Spanish veterinary centers were clinics and 7% hospitals. Significant differences were observed between age group and workplace frequencies of these professionals (χ^2^ = 15.187; *p* = 0.001), with a significantly lower percentage of veterinarians aged 24–34 working in clinics. As for female practitioners, again the youngest group (24–34 years) worked at a lower rate in hospitals (77.0%) (Fisher's exact test = 13.332; *p* = 0.001).

When asked about the species treated, 98.1% of veterinarians treated dogs; 96.5% cats, and only 36.7% small animals other than dogs and cats (rabbits, rodents, birds and reptiles). As many companion animal practitioners do not usually treat only one animal species, they were grouped into those who treated these other animals (36.7%) and those who did not (63.3%). We observed that animals different from dogs and cats were 2.7 times (95% CI: 1.343–5.541) more likely to be treated in a clinic than in a hospital. According to the Spanish Ministry of Agriculture, Food and Environment, the dog is the favorite pet, present in 21.9 % of households, followed by the cat (8.2%). Other species, such as ferrets and reptiles, have only recently become popular as pet animals (32).

With respect to the use of medicinal plants in small animals, only 2.9% of participants had not heard about it. Of the remaining (97.1%), 83.1% were in favor of their use, 5.1% were against and 11.8% did not answer. Table 1 details the opinion of the practitioners on the use of medicinal plants, taking into account the workplace of these professionals. The main reasons argued for their use were their benefits and their advantages as adjuvant therapy (38.9%), as well as the awareness of the scientific evidence for this use (34.1%). Only a small number of participants (*n* = 16) gave reasons against this therapy, justifying it on the lack of evidence (68.7%) and the poor therapeutic results obtained (31.3%). In German-speaking countries, practitioners explained that the main advantages of phytotherapy were its scarce adverse reactions (3, 24) and the acceptance of these treatments by the owners (3), whereas the main disadvantage was the lack of information (3).

**Table 1:** Reasons for using or not medicinal plants among the Spanish small animal veterinarians (*n* = 268).

**Reasons for**	**Workplace of veterinarians**			***p-*value (Fisher's exact test)**
	**Clinic frequency (%)**	**Hospital frequency (%)**	**Total frequency (%)**	
**Using medicinal plants**				
Beneficial and may be used as adjuvant therapy	86 (38.4)	12 (42.8)	98 (38.9)	0.341
There is scientific evidence	77 (34.4)	9 (32.1)	86 (34.1)	
Has no adverse effects	27 (12.0)	1 (3.6)	28 (11.1)	
Natural origin	21 (9.4)	2 (7.2)	23 (9.2)	
Others	13 (5.8)	4 (14.3)	17 (6.7)	
**Not using medicinal plants**				
Lack of scientific evidence	8 (72.7)	3 (60.0)	11 (68.7)	0.516
Poor therapeutic outcomes	3 (27.3)	2 (40.0)	5 (31.3)	

Most veterinarians of those who have heard about medicinal plants admitted to using phytotherapy (80.3%). This value is slightly higher than that indicated by other authors (75%) (3, 24). Practitioners have prescribed natural products mainly in both dogs and cats (45.1%); only in dogs (39.8%) and, to a lesser extent, in the three groups of companion animals (dogs, cats and other animals) (14.8%).

Of these veterinarians who have never used medicinal plants as therapy (19.7%), they justified that this was due to either the lack of knowledge (75%) or rejection (25%). In this sense, Hahn et al. (3) observed that the lack of information was the main reason for those practitioners not using phytotherapy (65%).

With respect to the health problems treated with medicinal plants (Table 2), ~3 out of 4 veterinarians (*n* = 244) used herbal products on the musculoskeletal system (70.1%), and more than a half prescribed them in dermatological (57.4%) and gastrointestinal (51.6%) disorders. These results are in accordance with other studies (6, 7, 33, 34). Pain and inflammations, gastrointestinal and skin troubles are usually related to non-severe diseases, and treated frequently with phytotherapeutic remedies. In the survey of Zitterl et al. (24) the most consumed phytomedicines were against digestive, pain and skin disorders. Hahn et al. (3) observed that practitioners prescribed herbal products usually in chronic disorders, and more than 67% employed them to prevent illnesses or treat those ones when conventional treatments do not work. Moreover, more than 50% of them used phytotherapy against joint and dermatological problems. According to Zitterl et al. (24), 75% of veterinarians used herbal products in acute diseases, 45% in chronic disorders and 50% as prophylactic therapy.

**Table 2:** Categories of health problems treated with natural products by the Spanish small animal veterinarians (35).

**Health problem**	**Frequency (%) (*n* = 244)**
Musculoskeletal	171 (70.1)
Dermatological	140 (57.4)
Gastrointestinal	126 (51.6)
Genitourinary	117 (48.0)
Respiratory	102 (41.8)
Behavioral disorders	101 (41.4)
Nervous	89 (36.5)
Parasitic	55 (22.5)
Oncologic	48 (19.7)
Cardiovascular	21 (8.6)
Others	23 (9.4)

In accordance with Wynn and Fougère, the main reason to start working with herbal medicines is the absence of answer to conventional treatments or if these ones are not available. This usually encompasses chronic processes such as immune-mediated diseases (atopic dermatitis, chronic bronchitis, chronic allergic rhinitis), tumors or digestive disorders (especially diarrhea), among others (22).

A detailed list of the medicinal plants reported by the veterinarians in the survey are shown in Tables 3, 4. Cannabis (70.5%) and Aloe (63.1%) were the most commonly prescribed natural products, followed by thyme (43.4%), artemisia and milk thistle (41% each one). In the end of this list, some veterinarians suggested other medicinal plants (18.4%) such as *Gingko biloba* and *Chamaemelum nobile* (1.4%) or *Echinacea* spp. (0.8%) (Table 4). Moreover, the characteristics of the top 15 medicinal plants indicated by practitioners are described in Table 5, including uses for disease, animal treated, if they are present in a monoherbal or polyherbal preparation and pharmaceutical form employed, as well as references of other authors who have previously reported its use in Spain or adjoining geographical regions. These medicinal plants were used to treat a wide range of diseases, and most of the preparations are intended for dogs and cats. On the other hand, approximately half of the commercial preparations are monoherbal and half polyherbal.

**Table 3:** Natural products commonly employed in disease treatment of the pets by Spanish small animal veterinarians.

**Natural products**	**Frequency (%) (*n* = 244)**
Cannabis (*Cannabis sativa*)	172 (70.5)
Aloe (*Aloe vera*)	154 (63.1)
Thyme (*Thymus vulgaris*)	106 (43.4)
Artemisia (*Artemisia annua*)	101 (41.4)
Milk thistle (*Silybum marianum*)	100 (41.0)
Bush clover (*Lespedeza capitata*)	98 (40.2)
Devil's claw (*Harpagophytum procumbens*)	88 (36.1)
Turmeric (*Curcuma longa*)	73 (29.9)
Calendula (*Calendula officinalis*)	71 (29.1)
Indian frankincense (*Boswellia serrata*)	65 (26.6)
Broadleaved pepperweed (*Lepidium latifolium*)	52 (21.3)
Rosemary (*Rosmarinus officinalis*)	46 (18.9)
Artichoke (*Cynara scolymus*)	46 (18.9)
Cranberry (*Vaccinium macrocarpum*)	17 (7.0)
Turkey tail (*Coriolus versicolor*)	10 (4.1)
Others	45 (18.4)

**Table 4:** Other natural products employed in disease treatment of the pets by Spanish small animal veterinarians.

**Natural products**	**Frequency (%) (*n* = 244)**
*Chamaemelum nobile*	3 (1.4)
*Gingko biloba*	3 (1.4)
*Echinacea purpurea*	2 (0.8)
*Ganoderma lucidum* ^*^	2 (0.8)
Honey^*^	2 (0.8)
*Allium sativum*	1 (0.4)
*Arctium lappa*	1 (0.4)
*Artemisia dracunculus*	1 (0.4)
*Arthrospira* spp.	1 (0.4)
*Betula péndula*	1 (0.4)
*Borago officinalis*	1 (0.4)
*Buddleja globosa*	1 (0.4)
*Calluna vulgaris*	1 (0.4)
*Crataegus monogyna*	1 (0.4)
*Eleutherococcus senticosus*	1 (0.4)
*Illicium verum*	1 (0.4)
*Malva sylvestris*	1 (0.4)
*Medicago sativa*	1 (0.4)
*Olea europaea*	1 (0.4)
*Passiflora incarnata*	1 (0.4)
*Petroselinum crispum*	1 (0.4)
*Peumus boldus*	1 (0.4)
*Piper aduncum*	1 (0.4)
*Primulae radix*	1 (0.4)
*Ribes nigrum*	1 (0.4)
Saccharomyces cerevisiae^*^	1 (0.4)
*Salvia officinale*	1 (0.4)
*Sambucus nigra*	1 (0.4)
*Scutellaria baicalensis*	1 (0.4)
*Strychnos nux-vomica*	1 (0.4)
*Symphytum officinale*	1 (0.4)
*Tilia europaea*	1 (0.4)
*Urtica dioica*	1 (0.4)
*Vaccinium myrtillus*	1 (0.4)
*Valeriana officinalis*	1 (0.4)
*Vanilla planifolia*	1 (0.4)
*Withania somnifera*	1 (0.4)
*Zingiber officinale*	1 (0.4)

* Not the product of medicinal plant.

**Table 5:** List of the top 15 natural products commonly employed by Spanish small animal veterinarians, along with their use, animal treated, type of preparation, pharmaceutical form and references of previous studies describing its use.

**Natural products**	**Use**	**Animal treated**	**Type of preparation**	**Pharmaceutical forms**	**References**
*Aloe vera*	Gastrointestinal and skin diseases	Dogs, cats	M/P	Cream/ointment, enema, shampoo, tablets	(10)
*Artemisia annua*	Immunostimulant, gastrointestinal diseases	Pets	M	Tablets	(36, 37)
*Boswellia serrata*	Inflammatory and osteoarticular disorders	Dogs, cats	P	Tablets	
*Calendula officinalis*	Wounds and skin diseases	Dogs, cats	P	Cream/ointment, shampoo	(3, 24, 36–38)
*Cannabis sativa*	Anxiety, cognitive dysfunction, cronic pain, inflammation	Dogs, cats	M	Oil, tablets	
*Coriolus versicolor*	Immunostimulant	Dogs, cats	M	Tablets	
*Curcuma longa*	Gastrointestinal, inflammatory and osteoarticular disorders	Dogs, cats	M/P	Powder, tablets	
*Cynara scolymus*	Hepatic diseases	Dogs, cats	P	Tablets	(3, 24, 36, 37)
*Harpagophytum procumbens*	Inflammatory and osteoarticular disorders	Dogs, cats	P	Tablets	(3, 24)
*Lepidium latifolium*	Urinary diseases	Dogs, cats	M	Drops	
*Lespedeza capitata*	Urinary diseases	Dogs, cats	M	Tablets	(3)
*Rosmarinus officinalis*	Gastrointestinal diseases, parasite repellent	Dogs, cats	M	Decoction/tisane, drops,	(10)
*Silybum marianum*	Hepatic diseases	Dogs, cats	P	Tablets, granulated sachets	(3, 24, 36, 37)
*Thymus vulgaris*	Respiratory diseases	Dogs, cats, rabbits, rodents, birds	M	Decoction/tisane, syrup	(3, 36, 37)
*Vaccinium macrocarpum*	Urinary diseases	Dogs	P	Tablets	

M, monoherbal; P, polyherbal.

As for *Cannabis sativa*, its leaves contain numerous compounds that may improve the quality of life in oncology patients, due to their analgesic, antiemetic, orexigenic, anxiolytic and antidepressant actions, and minimize the toxicity of conventional antineoplastic and anti-inflammatory treatments (39). All these properties may explain its current use in chronic processes.

In the case of *Aloe vera*, this herbal product is commonly employed in veterinary medicine for the treatment of digestive, dermatological, and ophthalmic diseases. Aloe juice has laxative effects, as it stimulates colon motility (40). Topical gel also has healing properties, as it accelerates the healing of wounds by stimulating the activity of macrophage and fibroblast activity (41), although other mechanisms are also implicated, such as hydration, insulation and protection (42). It inhibits thromboxane A2, a mediator of progressive tissue damage in pressure ulcers (41) and burns (43), and it is also angiogenic (44). In addition, it is effective in allergies, eczema, abscesses, fungal infections, pyodermas, and many types of dermatitis (45). Finally, it is also an interesting therapeutic option in ophthalmic disorders, against inflammation and infection in external parts of the eye (conjunctiva, lacrimal sac, cornea, and edges of the eyelids) (46).

As for *Thymus vulgaris*, the active compound present in the essential oil exhibits anthelmintic, antibacterial and antifungal properties (47), as well as antispasmodic activity (48). Moreover, it may be useful against bronchospasm (49) and as mucolytic (50), which may explain its wide use in chronic bronchitis.

Regarding pharmaceutical forms commonly employed in phytotherapy (Table 6), tablets (81.6%), syrup (oral or topical) (63.5%) and cream/ointment (53.7%) were the most frequently prescribed.

**Table 6:** Types of pharmaceuticals forms used for treatment of the pets by Spanish small animal veterinarians.

**Pharmaceutical forms**	**Frequency (%) (*n* = 244)**
Tablets (e.g., *Silybum marianum, Lespedeza capitata*)	199 (81.6)
Syrup (e.g., *Thymus vulgaris*)	155 (63.5)
Cream/ointment (e.g., *Aloe vera, Calendula officinalis*)	131 (53.7)
Oil (e.g., *Cannabis sativa*)	72 (29.5)
Shampoo (e.g., *Aloe vera, Calendula officinalis*)	45 (18.4)
Decoction/tisane (e.g., *Chamaemelum nobile, Thymus vulgaris*)	17 (7.0)
Enema (e.g., *Aloe vera*)	10 (4.1)
Others (e.g., *Lepidium latifolium, Silybum marianum*)	22 (9.0)

Tablets are quite useful, due to their good bioavailability, but also because some medicinal plants reduce palatability when added to animal feed. Therapeutic adherence and patient comfort should also be taken into account, as they are a simple option for customers (22). They also facilitate the administration of the exact dose, and it is the dosage form with the highest stability. As for syrups, they neutralize unpleasant tastes of the active ingredients, and their viscosity favors the contact with oral mucosa (22). Finally, ointments/creams are useful to treat acute or chronic dermatological disorders (22).

No significant differences were found between the number of disorders treated and the gender or workplace of the veterinarians (Mann-Whitney *U* test; *p* > 0.05) nor for the age group (Kruskal-Wallis test; *p* > 0.05), and the same happened with the number of natural products and the pharmaceutical forms used (Mann-Whitney *U* test; *p* > 0.05). However, specialists in dogs used significantly less natural products than those treating both dogs and cats, or the three groups of animals (dogs, cats and other animals) (Kruskal-Wallis test; *p* < 0.001).

As shown in Table 7, health problems treated with herbal medicines are not similar among the different species of pets. Musculoskeletal problems were the most commonly disorders treated with herbal products in dogs (71%) and cats (75.7%), whereas gastrointestinal diseases (83.3%) were the most frequent in pet animals other than dogs and cats. Something similar occurs with the natural products used, as in dogs (71%) and cats (79.3%) Cannabis was the most prescribed herbal product, and Aloe (80.6%) in other animals. Finally, tablets were always the most commonly used pharmaceutical forms in the three types of small animals.

**Table 7:** Frequency of most common health problems treated, natural products and pharmaceutical forms used by the Spanish small animal veterinarians.

**Species**	**Health problem**	**Natural products**	**Pharmaceutical forms**
		**Percentage**	
Dogs	Musculoskeletal 71.0	Cannabis 71.0	Tablets 82.4
	Dermatological 58.8	Aloe 63.9	Syrup 69.3
	Gastrointestinal 51.0	Thyme 44.1	Cream/ointment 54.2
Cats	Musculoskeletal 75.7	Cannabis 79.3	Tablets 87.9
	Gastrointestinal 68.6	Aloe 75.7	Syrup 75.0
	Dermatological 66.4	Thyme 55.7	Cream/ointment 62.9
Others^†^	Gastrointestinal 83.3	Aloe 80.6	Tablets 88.9
	Musculoskeletal 69.4	Cannabis 69.4	Cream/ointment 80.6
	Respiratory 69.4	Milk thistle 66.7	Syrup 75.0
	Dermatological 69.4		

† Small animals other than dogs and cats.

Little is known on the association of different sociodemographic variables of veterinarians with the pattern of use of medicinal plants. Table 8 displays the multivariate logistic regression analysis performed. Gender and workplace of the practitioners had a significant impact on the use of phytotherapy, as being female and working in a clinic the factors that increased the use of this type of treatment in 2.5 and 3.6 times, respectively. Older ages and treating animals like rabbits, rodents, birds and reptiles raised also the use of natural products, although no significant differences were revealed. Other researchers have also observed that phytotherapy was also better accepted among older professionals (41–60 years) (24). Regarding those professionals treating other animals different from dogs and cats, probably they are more familiar with medicinal plants as there are virtually no conventional treatments available for them. As pointed out by Zhang et al. (51), the use of complementary medicine in females may be related to a less mechanistic view of healing and disease, and to female social concept of caretaking. As for the influence of the workplace, for reasons of proximity, clinics are the most frequent place where animals are taken for consultation and treatment. In addition, and compared to hospitals, the disorders to be treated tend to be less severe, which in turn favors the use of phytotherapy.

**Table 8:** Multivariate logistic regression analysis showing the association between predictor variables and the use of phytotherapy (reference category: non-use phytotherapy).

**Predictor variables for use of phytotherapy**		**OR**	**95% CI Exp (B)**
Gender	Female	2.469	1.284–4.746^*^
Age	35–49	1.534	0.820–2.870
	50–64	1.484	0.644–3.423
Workplace	Clinic	3.601	1.650–7.858^*^
Species	Others^†^	1.402	0.768–2.559

* Significant differences (p ≤ 0.05).

† Small animals other than dogs and cats.

The present study also supports the need for a wide discussion on this topic, as it has highlighted the lack of academic training by the practitioners in this field. Our findings revealed that 97.1% of the participants admitted having heard of phytotherapy, but many health professionals do not receive university education on this subject. In fact, in Spain only pharmacists receive academic training on medicinal plants, and regarding other countries it is not easy to know if this topic is included in academic curricula. Stanossek and Wehrend have pointed out that complementary medicine may be part of elective courses in some German universities (52). This contrasts with the growing importance of herbal medicines in Europe (53, 54), even though it is conducted mostly in developing countries (54). Traditional knowledge of medicinal plants in animals has been recorded in 12 out of 37 European Union and associated countries, being Italy, Spain and Turkey where most research has been carried out (53). In Southern Europe (2, 55) and in particular in Iberian Peninsula, specific work in this field is scarce (8–10, 34, 56, 57). As for Northern Europe, in a survey among veterinarians in German-speaking countries (Austria, Germany and Switzerland), Hahn et al. (3) showed that 79% of 189 participants used medicinal plants in small animal medicine. In the study of Truls (58), over 60% of the 64 veterinarians in Austria employed herbal medicine as therapeutic option. Ertl (59) described that 76% of the 36 practitioners in Kärnten (Austria) used phytotherapy against acute diseases. In an American retrospective study from 2005, Shmalberg and Memon (60) revealed that 39% of 5,195 pets were treated with integrative treatment modalities (neither homeopathy nor chiropractic treatments were offered) at the Small Animal Clinical Science (University of Florida).

One of the major obstacles to carrying out this therapy is not only the lack of support from clients, but also from other veterinarians. Money and time also hinder its practice, as it does not generate the same income as conventional therapy, and is time-consuming to develop. Moreover, finding reliable information on the appropriate medicinal plants or master formulas specifically targeted to animals is difficult (22). In this regard, there is a database available on the Internet, belonging to the Institute of Veterinary Pharmacology and Toxicology in Zurich, which collects information about the most commonly used medicinal plants, their toxicity, potential interactions and targeted pathologies, in both pets and rental animals, as well as their appropriate dosage regimen (61).

Several limitations must be acknowledged. The voluntary nature of participation may bias the study, as only veterinarians specially interested in phytotherapy could only have taken part. As data were collected through on line, there was no direct interaction with the respondents, and sometimes the authors had to group the information provided, especially in the two open-ended questions. On the other hand, although it was requested that practitioners with <1 year experience should not take part, we have no way of verifying this. Finally, women proportion was higher than that of men, which should also be taken into account. Despite these inherent limitations of the survey, the results increase our understanding on the use of herbal medicines in veterinary practice, as well as help to define the pattern of those practitioners prescribing this therapy.

Up to the best of our knowledge, this is the first study that aims to describe the use of medicinal plants by veterinarians in small animal medicine in Spain. We have demonstrated that the use of medicinal plants is wide among the population surveyed (small animal practitioners). Only a small proportion of professionals had not heard of it, and did not use it due to the lack of information. Medicinal plants are mainly used by female veterinarians, in clinics and in both dogs and cats. Cannabis, Aloe and Thyme were the most employed medicinal plants against musculoskeletal, dermatological and digestive disorders.

In conclusion, in this study we have documented the use, patterns and attitudes of small animal veterinarians toward medicinal plants in Spain, as well as those factors that may influence the choice of this type of treatment. Widespread use of medicinal plants has been demonstrated, and most veterinarians also showed a positive attitude toward herbal medicines. A high proportion of practitioners who took part in the survey have used this type of therapy, mainly against musculoskeletal, dermatological and digestive diseases. The most common pattern of user among those veterinarians surveyed was women working in clinics. Of those who had never used medicinal plants, the majority attributed this to a lack of training in this field. Further studies should be conducted to assess the most commonly prescribed herbal medicines for each disorder and species of pet animal, as well as the actual efficacy of these treatments compared to modern medicine, in order to improve evidence-based practices in this therapy. The wide use of phytotherapy would also justify the need for a debate on the source of veterinarians' knowledge in this field and the lack of academic training provided by veterinary faculties.

## Data availability statement

The original questionnaire presented in the study are included in the article/Supplementary material, further inquiries can be directed to the corresponding author.

## Ethics statement

The studies involving human participants were reviewed and approved by the study was approved in advance by the Ethics Committee of the University of León (ULE0382018). The patients/participants provided their written informed consent to participate in this study.

## Author contributions

BR and RD designed the study and the methodology. JS carried out the formal analysis. MD and CL performed data curation. BR wrote the original draft manuscript. AS and NF reviewed and edited the final manuscript. JG and MS were responsible for project administration. All authors contributed to the article and approved the submitted version.

## References

[1] Köhler-Rollefson I Bräunig J. Antrophological veterinary medicine: the need for indigenizing the curriculum. In: 9th International Conference of Institutions of Tropical Vetereinary Medicine. Harare, Zimbabwe (1998).

[2] Viegi L Pieroni A Guarrera PM Vangelisti R. A review of plants used in folk veterinary medicine in Italy as basis for a databank. J Ethnopharmacol. (2003) 89:221–244. 10.1016/j.jep.2003.08.003 14611886

[3] Hahn I Zitterl-Eglseer K Franz C. Phytomedizin bei Hund und Katze: Internetumfrage bei Tierärzten und Tierärztinnen in Österreich, Deutschland und der Schweiz. Schweiz Arch Tierheilkd. (2005) 147:135–41. 10.1024/0036-7281.147.3.135 15801625

[4] Mayer M Zbinden M Vogl CR Ivemeyer S Meier B Amorena M. Swiss ethnoveterinary knowledge on medicinal plants: a within-country comparison of Italian speaking regions with north-western German speaking regions. J Ethnobiol Ethnomed. (2017) 13:1. 10.1186/s13002-016-0106-y 28049539 PMC5209851

[5] Bullitta S Re GA Manunta MDI Piluzza G. Traditional knowledge about plant, animal, and mineral-based remedies to treat cattle, pigs, horses, and other domestic animals in the Mediterranean island of Sardinia. J Ethnobiol Ethnomed. (2018) 14:50. 10.1186/s13002-018-0250-7 30029686 PMC6054737

[6] Marković MS Pljevljakušić DS Nikolić BM Miladinović DL Djokić MM Rakonjac LB. Ethnoveterinary knowledge in Pirot County (Serbia). S Afr J Bot. (2021) 137:278–89. 10.1016/j.sajb.2020.10.025

[7] Mattalia G Belichenko O Kalle R Kolosova V Kuznetsova N Prakofjewa J. Multifarious trajectories in plant-based ethnoveterinary knowledge in Northern and Southern Eastern Europe. Front Vet Sci. (2021) 8:710019. 10.3389/fvets.2021.710019 34722694 PMC8551763

[8] Agelet A Valles J. Vascular plants used in ethnoveterinary in Pallars (Pyrenees, Catalonia, Iberian Peninsula). In:Pieroni A, editor. Herbs, Humans and Animals. Köln: Experiences Verlag (1999). p. 14–35.

[9] Bonet MÀ Vallès J. Ethnobotany of Montseny biosphere reserve (Catalonia, Iberian Peninsula): plants used in veterinary medicine. J Ethnopharmacol. (2007) 110:130–47. 10.1016/j.jep.2006.09.016 17059874

[10] Carrió E Rigat M Garnatje T Mayans M Parada M Vallès J. Plant ethnoveterinary practices in two pyrenean territories of catalonia (Iberian Peninsula) and in two areas of the balearic islands and comparison with ethnobotanical uses in human medicine. Evid Based Compl Alternat Med. (2012) 2012:896295. 10.1155/2012/896295 22829861 PMC3399547

[11] González JA Vallejo JR. Relics and historical uses of human zootherapeutic products in contemporary Spanish ethnoveterinary medicine. Vet Sci. (2021) 8:323. 10.3390/vetsci8120323 34941850 PMC8707080

[12] Kubkomawa HI Nafarnda DW Tizhe MA Daniel TK Shua NJ Ugwu CC. Ethno-veterinary health management practices amongst livestock producers in Africa: a review. Adv Agric Sci. (2020) 6:1–006.

[13] Maldonado C Paniagua-Zambrana N Bussmann W R Zenteno-Ruiz FS Fuentes A. La importancia de las plantas medicinales, su taxonomía y la búsqueda de la cura a la enfermedad que causa el coronavirus (COVID-19). Ecol Bol. (2020) 55:1–5.

[14] Shao Y Wang Y Yuan Y Xie Y. A systematic review on antibiotics misuse in livestock and aquaculture and regulation implications in China. Sci Total Environ. (2021) 798:149205. 10.1016/j.scitotenv.2021.149205 34375247

[15] Reig M Toldrá F. Veterinary drug residues in meat: concerns and rapid methods for detection. Meat Sci. (2008) 78:60–7. 10.1016/j.meatsci.2007.07.029 22062096

[16] Dominic AT Sriamarao P Cardona C Sterr CJ Kennedy S Sreevatsan S. One medicine one science: a framework for exploring challenges at the intersection of animals, humans, and the environment. Ann N Y Acad Sci. (2014) 1334:26–44. 10.1111/nyas.12601 25476836 PMC4383647

[17] Rock M Buntain BJ Hatfield JM Hallgrímsson B. Animal–human connections, “one health,” and the syndemic approach to prevention. Soc Sci Med. (2009) 68:991–5. 10.1016/j.socscimed.2008.12.047 19157669

[18] Schwabe C. Veterinary medicine and human health. 3rd ed. Baltimore: Williams & Wilkins (1964). p. 1–680.

[19] Zinsstag J Mackenzie JS Jeggo M Heymann DL Patz JA Daszak P. Mainstreaming one health. Ecohealth. (2012) 9:107–10. 10.1007/s10393-012-0772-8 22777051 PMC3415611

[20] Luo B Hu Q Lai K Bhatt A Hu R. Ethnoveterinary survey conducted in Baiku Yao communities in Southwest China. Front Vet Sci. (2022) 8:813737. 10.3389/fvets.2021.813737 35146017 PMC8822042

[21] Rivera D Verde A Fajardo Rodríguez J Ríos S Alcaraz F Cárceles C. Ethnoveterinary medicine and ethnopharmacology in the main transhumance areas of Castilla-La Mancha (Spain). Front Vet Sci. (2022) 9:866132. 10.3389/fvets.2022.866132 35591874 PMC9113055

[22] Wynn S Fougère B. Clinical practice: getting started. In: Wynn S, Fougère B, editors. Veterinary Herbal Medicine. St. Louis: Mosby Elsevier (2006). p. 453–457. 10.1016/B978-0-323-02998-8.50027-5

[23] Shmalberg J Xie H Memon MA. Canine and feline patients referred exclusively for acupuncture and herbs: a 2-year retrospective analysis. JAMS J Acupunct Merid Stud. (2019) 12:160–5. 10.1016/j.jams.2019.04.002 31028973

[24] Zitterl-Eglseer K Truls C Munoz Vinent LR Ertl M Kern M Zitterl W. Umfrage über den Einsatz von pflanzlichen Arzneimitteln in Tierarztpraxen in Österreich. Vet Med Aust. (2004) 91:236–41.

[25] Stevenson MA. Sample size estimation in veterinary epidemiologic research. Front Vet Sci. (2021) 7:539573. 10.3389/fvets.2020.539573 33681313 PMC7925405

[26] O'Connor AM Sargeant JM Dohoo IR Erb HN Cevallos M Egger M. Explanation and elaboration document for the STROBE-vet statement: strengthening the reporting of observational studies in epidemiology—veterinary extension. J Vet Intern Med. (2016) 30:1896–928. 10.1111/jvim.14592 27859752 PMC5115190

[27] Boletín Oficial del Estado. Disposición 16673 del BOE núm. 294 de 2018. (2018). Available online at: http://www.boe.es (accessed October 1, 2022).

[28] McDonald RP. Test Theory. A Unified Treatment. New York, NY; Mahwah NJ: Lawrence Erlbaum Associates (1999).

[29] Instituto Nacional de Estadística. Veterinarios Colegiados por año y Sexo. Madrid, Spain (2020). Available online at: https://www.ine.es/jaxi/Datos.htm?tpx=30722 (accessed September 21, 2022).

[30] Instituto Nacional de Estadística. Veterinarios por Comunidades, Ciudades Autónomas y Provincias de Colegiación, Edad y Sexo. Madrid, Spain (2020). Available online at: https://www.ine.es/jaxi/Datos.htm?tpx=48567 (accessed September 21, 2022).

[31] Veterinary Management Studies. La Clínica Veterinaria Española. Lérida, Spain (2019). Available online at: https://www.estudiosveterinarios.com/home.aspx#2 (accessed April 21, 2022).

[32] Ministerio de Agricultura A y MA. Análisis y caracterización del sector de los animales de compañía. Madrid, Spain (2015), 1–75. Available online at: https://www.mapa.gob.es/es/ganaderia/temas/produccion-y-mercados-ganaderos/20160222_informeestudioparapublicar_tcm30-104720.pdf (accessed September 21, 2022).

[33] Parada M Carrió E Bonet MÀ Vallès J. Ethnobotany of the Alt Empordà region (Catalonia, Iberian Peninsula). Plants used in human traditional medicine. J Ethnopharmacol. (2009) 124:609–18. 10.1016/j.jep.2009.04.050 19422899

[34] Benítez G González-Tejero MR Molero-Mesa J. Knowledge of ethnoveterinary medicine in the Province of Granada, Andalusia, Spain. J Ethnopharmacol. (2012) 139:429–39. 10.1016/j.jep.2011.11.029 22155471

[35] Cook F. Economic Botany Data Collection Standard. Kew: Royal Botanic Gardens (1995).

[36] Russo R Autore G Severino L. Pharmaco-toxicological aspects of herbal drugs used in domestic animals. Nat Prod Commun. (2009) 4:1777–84. 10.1177/1934578X0900401230 20120122

[37] Severino L Russo R Autore G Marzocco S de Tommasi N. Use of phytotherapics in dogs and cats. Pharmacologyonline. (2008) 2:12–21.

[38] Tresch M Mevissen M Ayrle H Melzig M Roosje P Walkenhorst M. Medicinal plants as therapeutic options for topical treatment in canine dermatology? A systematic review. BMC Vet Res. (2019) 15:174. 10.1186/s12917-019-1854-4 31133058 PMC6537371

[39] Tejada R. Cannabis en Veterinaria: Producto Fitoterápico. Aplicaciones en pacientes oncológicos. Madrid: Cannabis en Veterinaria (2020).

[40] de Witte P. Metabolism and pharmacokinetics of anthranoids. Pharmacology. (1993) 47:86–97. 8234447 10.1159/000139847

[41] Davis R Leitner M Russo J Byrne M. Wound healing. Oral and topical activity of Aloe vera. J Am Podiatr Med Assoc. (1989) 79:559–62. 2607423 10.7547/87507315-79-11-559

[42] Bruneton J. Pharmacognosy, Phytochemistry, Medicinal Plants. Paris: Lavosier (1995).

[43] Swain S Lee A. Topical wound medications: a review. J Am Vet Med Assoc. (1987) 190:1588–93. 3301764

[44] Moon E Lee Y Lee O Lee M Lee S Chung M. A novel angiogenic factor derived from Aloe vera gel: beta-sitosterol, a plant sterol. Angiogenesis. (1999) 3:117–23. 14517429 10.1023/a:1009058232389

[45] Wynn S Fougère B. Materia medica. In: Wynn S, Fougère B, editors. Veterinary Herbal Medicine. St. Louis: Mosby Elsevier (2006). p. 459–672. 10.1016/B978-0-323-02998-8.50028-7

[46] Kodym A Grześkowiak E Partyka D Marcinkowski A Kaczyńska-Dyba E. Biopharmaceutical assessment of eye drops containing aloe (Aloe arborescens Mill) and neomycin sulphate. Acta Pol Pharm. (2002) 59:181–6. 12230244

[47] Maybe R. The Complete New Herbal. London: Elm Tree Books (1988).

[48] Cruz T Jimenez J Zarzuelo A Cabo M. The spasmolytic activity of the essential oil of Thymus baeticus Boiss in rats. Phytotherapy Res. (1989) 3:106–8.

[49] Bisset N Wichtl M. Herbal Drugs and Phytopharmaceuticals. Boca Raton: CRC Press (1994).

[50] van den Broucke C Lemli J. Spasmolytic activity of the flavonoids from Thymus vulgaris. Pharm Weekbl Sci. (1983) 5:9–14. 6844124 10.1007/BF01959645

[51] Zhang Y Leach MJ Hall H Sundberg T Ward L Sibbritt D. Differences between male and female consumers of complementary and alternative medicine in a national US population: a secondary analysis of 2012 NIHS data. Evid Based Complement Alt Med. (2015) 2015:413173. 10.1155/2015/413173 25861360 PMC4377351

[52] Stanossek I Wehrend A. Application of veterinary naturopathy and complementary medicine in small animal medicine: a survey among German veterinary practitioners. PLoS ONE. (2022) 17:e0264022. 10.1371/journal.pone.0264022 35226679 PMC8884514

[53] Mayer M Vogl CR Amorena M Hamburger M Walkenhorst M. Treatment of organic livestock with medicinal plants: a systematic review of European ethnoveterinary research. Compl Med Res. (2014) 21:375–86. 10.1159/000370216 25592949

[54] Katerere D Luseba D editors. Ethnoveterinary Botanical Medicine: Herbal Medicines for Animal Health. Boca Raton: CRC Press (2010). p. 1–450. 10.1201/EBK1420045604

[55] Pieroni A. Herbs, Humans and Animals/Erbe, uomini e bestie. Köln: Experiences Verlag (1999).

[56] Belda A Martínez-Pérez JE Martín C Peiró V Seva E. Plants used to capture and sustain wild finches (Fringillidae) in Southeast Spain. Econ Bot. (2010) 64:367–73. 10.1007/s12231-010-9129-922611428

[57] Akerreta S Calvo MI Cavero RY. Ethnoveterinary knowledge in Navarra (Iberian Peninsula). J Ethnopharmacol. (2010) 130:369–78. 10.1016/j.jep.2010.05.023 20573568

[58] Truls C. Einsatz von pflanzlichen Arzneien in der Kleintierpraxis (dissertation thesis). Vienna: Veterinary University (1999).

[59] Ertl M. Einsatz von pflanzlichen Arzneien in Tierarztpraxen in Kärnten (dissertation thesis). Vienna: Veterinary University (2002).

[60] Shmalberg J Memon MA. A retrospective analysis of 5,195 patient treatment sessions in an integrative veterinary medicine service: patient characteristics, presenting complaints, and therapeutic interventions. Vet Med Int. (2015) 2015:983621. 10.1155/2015/983621 26798552 PMC4699059

[61] Institut für Veterinärpharmakologie und Toxikologie. CliniPharm/ClinTox. Zürich, Germany (2020). Available online at: https://www.vetpharm.uzh.ch/cpthome.htm (accessed April 25, 2022).

