# Supplementary material for: Use of medicinal plants by veterinary practitioners in Spain: A cross-sectional survey
Source: Front Vet Sci. 2022 Dec 15;9:1060738. doi: 10.3389/fvets.2022.1060738 (PMC9797804; doi:10.3389/fvets.2022.1060738)
Supplement: Supplementary file 1 [file Data_Sheet_1.docx]

1. Gender.

 Female  Male

2. Age.

 24-34  35-49  50-64  <65

3. Workplace.

| Veterinary clinic |  |
| --- | --- |
| Veterinary hospital |  |

4. Province.

5. You provide services to...

 Dogs  Cats  Exotic animals

6. Have you ever heard about the use of Phytotherapy and natural products in veterinary medicine??

- Yes (Please, go to question 8)
- No (Please, go to question 7)

7. Would you like to receive information about?

 Yes  No

| **VETERINARY PHYTOTHERAPY** |  | **VETERINARY PHYTOTHERAPY** |
| --- | --- | --- |

8. Are you for or against its use? Please, explain why.

9. Have you ever used Phytotherapy or natural products for your patients?

- Yes (Please, go to question 10)
- No (Please, go to question 15)

10. In which patients have you used it?

 Dogs  Cats  Exotic animals

11. Please, select those health problems you have treated with Phytotherapy.

Dermatological 

Musculoskeletal 

Nervous 

Respiratory 

Cardiovascular 

Gastrointestinal 

Genitourinary 

Oncologic 

Behavioural disorders 

Parasitic 

Others 

| 12. Which products have you used?  Aloe (*Aloe vera*)  Rosemary (*Rosmarinus officinalis*)  Calendula (*Calendula officinalis*)  Milk thistle (*Silybum marianum*)  Artichoke (*Cynara scolymus*)  Bush clover (*Lespedeza capitata*)  Turmeric (*Curcuma longa*)  Indian frankincense (*Boswellia serrata*)  Devil’s claw (*Harpagophytum procumbens*)  Thyme (*Thymus vulgaris*)  Cranberry (*Vaccinium macrocarpum*)  Broadleaved pepperweed (*Lepidium latifolium*)  Turkey tail (*Coriolus versicolor*)  Cannabis (*Cannabis sativa*)  Artemisia (*Artemisia annua*)  Others | | | |                                | |  |
| --- | --- | --- | --- | --- | --- | --- |
|  |  | |  |  |  |  |
| 13. What dosage form do you usually use to  administer these products? | | | | | | |
| Syrup |  |  |  |  |  |  |
| Tablet |  |  |  |  |  |  |
| Enema |  |  |  |  |  |  |
| Oil |  |  |  |  |  |  |
| Ointment/Cream |  |  |  |  |  |  |
| Shampoo |  |  |  |  |  |  |
| Others  | | | | |  | |

14. Was the treatment successful?

15. Why have you not used them?
